# Increased social differences, loneliness, and sleep deprivation among undergraduate nursing students in Norway, when writing their bachelor’s thesis during the COVID-19 pandemic. A qualitative study

**DOI:** 10.1177/20503121251360296

**Published:** 2025-08-23

**Authors:** Beate André, Guro Karlsholm, Kjersti Grønning

**Affiliations:** 1 Department of Public Health and Nursing, Norwegian University of Science and Technology, Trondheim, Norway; 2 St. Olav Hospital, Trondheim University Hospital, Torgarden, Norway; 3 Department of Research, Nord-Trøndelag Hospital Trust, Levanger, Norway

**Keywords:** Bachelor thesis, close-down, COVID-19, undergraduate nursing students, qualitative interviews

## Abstract

**Objectives:**

The outbreak of COVID-19 in 2020 resulted in closed universities, digital teaching and restricted social contact. The students may encounter psychological stress and worries about their careers ahead, and some may experience feelings of doubt and helplessness while studying. Little is known about this closure’s impact on undergraduate nursing students writing their bachelor’s theses. This study aimed to explore how undergraduate nursing students in Norway, writing their bachelor’s theses, experienced coping with the impact of COVID-19 on their social and academic lives.

**Methods:**

We used qualitative individual in-depth interviews, and 14 undergraduate nursing students participated. Data were analyzed using content analysis based on Brinkmann and Kvale.

**Results:**

The analyses showed that the social restrictions demanded that the students had to cope with several challenges related to their social and academic lives. Two main categories and four subcategories emerged during the analysis. The main categories were (1) Practical challenges, with Physical restrictions and digitalisation and Circadian rhythm and sleep deprivation as subcategories and (2) Mental challenges, with the sub-categories’ Social isolation and loneliness, and Motivational and emotional challenges.

**Conclusion:**

When the universities closed, the students struggled with loneliness, sleep deprivation, keeping their routines, and being motivated to complete their bachelor’s theses. The universities must develop strategies to ensure that the students receive satisfactory information, support and guidance.

## Introduction

Due to COVID-19, the universities had to change to digital teaching, and the universities in Norway were locked down. Digital teaching has several advantages.^1,2^ It is more flexible, and it enables students to work independently at their own learning pace. Digital teaching also has several disadvantages, such as a lack of social interaction, difficulties in retaining students’ attention, and inadequate instant feedback.^1^ It is also shown that the pandemic and the enforcement of stringent social distancing measures stirred negative emotions and disrupted the students’ usual lives profoundly.^3^ Before the pandemic, students were pursuing their academic goals while enjoying a life full of social interactions, but the closure of universities resulted in no physical courses or interaction. Both the teaching and semester examinations were online, causing considerable concerns among the students. The necessary closure of universities and the requirement to adapt to and cope with distance learning had the strongest impact on undergraduate students.^3^ Even though the COVID-19 pandemic has brought many issues to the foreground of nursing education,^4,5^ introducing all the advantages of digital teaching,^1^ it is important to keep in mind the importance of learning in a social context^6,7^ and how social distancing influences students’ mental health and loneliness.^8^ Loneliness may be defined as a circumstance in which a person encounters an individual shortage of social relationships, quantitatively or qualitatively.^9^ Lack of social relationships leads to social loneliness, which possibly be a distinct type of loneliness that may occur related to the lockdown based on the COVID-19 outbreak.^10^ Loneliness, in general, may affect a person’s health, and has earlier been associated with both depression and suicidal behaviour.^9,11^

In many countries across the world, the COVID-19 outbreak and social distancing influenced people’s mental health, quality of life, well-being and loneliness.^8^ Other studies show that age, gender, being single or living alone are found to be significant demographic determinants of loneliness in general,^9,12^ while anxiety, fear, sadness and anger are associated with reduced coping strategies among nursing students.^13^ According to Lazarus and Folkman,^14^ coping refers to a person’s responses aimed at managing a problem caused by stress. They categorise coping strategies as problem- or emotion-focused. Problem-focused strategies are active efforts made by the person to change a stressful situation, while emotion-focused strategies consist of changing the cognitive focus toward the stressor or the relational meaning of what is happening.^14^ Research shows that a lack of social interaction and contact with others challenges a person’s ordinary coping strategies and how the person usually deals with problems,^15^ while proactive coping involves gaining skills and abilities to assess the changing environment. However, when facing a pandemic, nursing students additionally undergo extreme psychological stress. They get concerned about their career ahead and may experience feelings of doubt and helplessness.^16,17^ It is shown that students often develop immature or negative coping strategies instead of positive problem-solving methods when faced with pressure caused by crises.^17^

Results from the first two waves of the Student Health and Well-being Survey (SHoT) in Norway show an increase in mental health problems among Norwegian college students from 2010 (16%) to (29%) in 2018.^18^ The study also reveals a slight decrease in the proportion of students reporting having a good quality of life compared to the previous SHoT surveys. Thirty-nine percent of students stated a good quality of life from 2010 to 2018, while this proportion in 2021 was 34%.^19^ In the same year, as many as 44% of the students reported that they often or very often missed someone to be with, 24% felt left out, while 37% often or very often felt isolated.^19^ A study conducted in Norway between 9 and 29 March showed that people were more concerned after the COVID-19 outbreak regulations were implemented. The groups expressing the greatest increase in the level of concern were single people under the age of 45 and immigrants,^20^ and nursing undergraduate students might be represented in all these groups.

Several studies have also shown that inadequate sleep duration (<8 h/day) is related to numerous harmful health outcomes such as hypertension, diabetes and the risk of suicide in adults.^21–24^ A study of Norwegian adolescents before the COVID-19 pandemic revealed that sleep duration was related to several health-risk behaviours, such as smoking, irregular meal patterns and poor academic achievement.^25,26^

A recent cross-sectional study among undergraduate nursing students at five Norwegian universities showed that the nursing students reported worse outcomes during the COVID-19 pandemic on general health, psychological distress and overall QoL compared to the reference population.^27^ It is also shown that an increased number of students reporting loneliness in nursing schools might have been attributed to the mandatory lockdown due to the coronavirus.^28^ The relationship between students and teachers was also weakened during the lock-down period of the pandemic when all teaching and contact between teachers and students were digitalised,^29^ which may be challenging for undergraduate nursing students when writing their bachelor thesis, as they depend on supervision from the teachers.^30,31^ However, in existing research, little is known about the impact this pandemic lock-down has on Norwegian undergraduate nursing students’ social and academic life when writing their bachelor’s thesis.

On this background, we wanted to explore how Norwegian undergraduate nursing students, writing their bachelor’s thesis, experienced coping with the impact of COVID-19 on their social and academic lives.

## Methods

### Setting

The setting for this study was the Faculty of Medicine, Department of Public Health and Nursing at Norwegian University of Science and Technology (NTNU). Approximately 200 undergraduate nursing students are educated each year, and the programme leads to a bachelor’s degree in nursing that qualifies for authorisation as a registered nurse in Norway. All students write their bachelor’s thesis as a final exam. The bachelor’s thesis in Norway usually constitutes 15 European Credit Transfer and Accumulation System credits and is the largest academic work students undertake after six semesters of study at the university level.^32^ The primary learning outcomes are related to nursing competence, nursing science and research methods.^33^ All students must formulate a research question, attend theoretical lectures and bachelor seminars and have mandatory individual supervision. This study took place in April 2020, at the start of the lockdown. The nursing students participating in this study were informed about the lockdown only days before the course started for the bachelor’s thesis, in March 2020.

### Recruitment

An information note was posted on the students’ digital platform, Blackboard, inviting them to participate in qualitative in-depth interviews. There were 170 eligible students. The post included information about the purpose of the study, topics for the interview, information about the researchers, and the practical organisation of the interviews were done digitally. The supervisors were also encouraged to remind their students about the posted invitation and their ability to participate in the study. The students were in their last semester and working on their final exam, the bachelor thesis. The students who were interested in participating in the study sent an email to the second author, who conducted the interviews, being a convenience sampling.

### Data collection

We collected the data through qualitative individual semi-structured in-depth interviews^34,35^ to explore how Norwegian undergraduate nursing students writing their bachelor thesis experienced coping with the impact of COVID-19 on their social and academic lives.

This study is part of a bigger study investigating what and how nursing students learn from writing their bachelor’s thesis.^33^ The data in this article is distinct from that in an earlier publication.^33^ The study’s aims, outcomes, findings and conclusions differ, ensuring that no data from nursing undergraduate students is used more than once.

Due to the COVID-19 restrictions, the interviews were conducted digitally, through a licensed version of Zoom. The students were sitting in their family homes or student homes and instructed to find a place where they would be undisturbed during the time of the interview. The interviews lasted approximately 45 min and were audio recorded and transcribed verbatim by the second author, an RN who was a PhD student at the time of the data collection. The interviewer did not have any relationships with the students before the study. The thematic interview guide was developed by all three authors and contained questions about how undergraduate nursing students experienced their student life in general while writing their bachelor’s theses, their daily routines, study techniques, and their motivation and ambitions. The main questions were open-ended, pursued by investigative sub-questions (e.g. please supply an example of this, do you have relevant information to add). Data saturation was obtained when 14 undergraduate nursing students had participated in the interview.^36^

### Data analysis

To get a deeper understanding of the informants’ experiences, we chose content analysis as our analysis strategy.^34,36,37^ The second author read and coded all interviews. To secure the confirmability of the material, all researchers reviewed and analysed the interview material.^38^ The authors discussed the meaning and the different interpretations of the statements from the informants before a consensus of interpretation was reached.^36^ To help with the systematising and coding of the transcriptions NVivo 12Pro was used.^39^ The analysis was finalised involving the process identified by Brinkmann and Kvale.^34^ First, the statements were detected in the transcriptions and condensed into meaning components. Then, the codes were separated from the meaning components, the categories were derived inductively from the data, and lastly, we selected the quotations. Examples of questions from the interview guide are: *Will you tell me about a typical day of studies during the BT?* and *What do you do to keep motivated during your work?* Table 1 shows an example of the analysis process.

**Table 1.** Example of analysis.

Statement	Coding	Subcategory	Main category
‘Then it is that I was losing the routines quite quickly when the pandemic came – I thought this was rather hopeless, I would not be able to do this – but when I managed to maintain the routines, it was better to write the bachelor thesis’ (7).	- Losing routines - Hopelessness - Maintain routines	Circadian rhythm and sleep deprivation	Practical challenges

### Ethical considerations

Participation in the study was voluntary. All students received oral and written information about the study’s purpose and gave written consent to participate via the Select survey. All data gathered were confidential. The Norwegian Centre for Research Data approved the study (NSD, ref no: 457038).

## Results

### Sample characteristics

The informants included 14 undergraduate nursing students at the Faculty of Medicine, Department of Public Health and Nursing at the NTNU in their third year of a 3-year programme. The students were between 22 and 27 years old, and the sample consisted of 12 female and 2 male students. Only two of the students had experience with higher education before entering the bachelor’s in nursing studies, but none had written a bachelor’s thesis before.

### Identified categories

The analyses showed that the students experienced coping with the COVID-19 lockdown as challenging, both for their social and academic lives. The results are presented in two main categories: (1) practical challenges and (2) mental challenges (Table 2). The findings are further elaborated with statements from the students. The informants are numbered in parentheses at the end of each statement.

**Table 2.** The identified categories.

Main categories	Subcategories
Practical challenges	Physical restrictions and digitalisation
	Circadian rhythm and sleep deprivation
Mental challenges	Social isolation and loneliness
	Motivational and emotional challenges

### Practical challenges

This category consists of statements about practical issues that made the student life situation with the pandemic difficult for the informants while working on their bachelor’s theses.

#### Physical restrictions and digitalisation

The lockdown resulted in the digitalisation of lectures, without any preparation.

After a while, I came up with digital lectures and such, but it was very difficult to follow – it was difficult to see those lectures – it would have been much easier without the coronavirus because then we would have been on campus. (12)

Both teaching and supervision were conducted on digital platforms, and no physical meetings were allowed. When all the learning activities were changed ‘overnight’, it was difficult for the students to keep up with all the changes. The students hoped that everything would go back to normal:

Yes, both the school and the library have been closed – so there has been no one to ask for help with literature searches and relevant articles -I have no one to ask – it has been a bit tiring. (3)

Several students described that the COVID-19 lockdown made it challenging to carry on with their studies because the library was closed. A closed library resulted in a lack of library services and difficulties in obtaining books and other literature that they needed for their bachelor’s theses. Some of the students were also unable to find help or did not know who to ask for support:

At the beginning it was very difficult to get hold of literature, curriculum literature, it was challenging, I had some books at home that I could use, but then there were several books that I missed, but then there was an open online version of some of the curriculum books, so that helped a lot, but I remember that was a very big challenge at the beginning. (5)

Several of the students used the library and the smaller rooms at the university for studying and socializing before the pandemic. The closing of the university made it difficult to continue their academic work with their bachelor’s theses and challenged their study routines, independence and control of the study situation:

But it’s just little things, become big because you’re sitting there and, yes, you’re a bit further away, we’ve been a bit further away from our tutors and our classmates in the whole process. (14)

The distance from both classmates and supervisors made the process of writing the bachelor’s thesis more difficult. Small issues became bigger in this situation because the students did not have classmates to discuss with, and they felt that the supervisors were far away and difficult to reach:

Without the pandemic then I would have spent a lot more time in the library, and maybe had a little more structured sessions and days, and then the supervisor would have been more accessible, I think at least, that it’s easier to just drop by the office or something like that, and then I would have had access to printouts, we could also have had all the teaching, and met as normal then, that I might, I think at least I would have gotten a little more out of the seminars then, but that’s it. (6)

#### Circadian rhythm and sleep deprivation

The students experienced it as difficult with the sudden change in all university activities, making it challenging to keep the study routines they used to have and to continue their academic work with their theses:

Without the pandemic that maybe it would have been a little easier to get up at the right time and sort of get to school because you’re going to meet someone, not necessarily to write each other’s assignments, but just sit together and write, and then maybe you’ve been exercising and then home and then had the evening in a way, you could have taken the evening off then, whereas when things have been like now it’s a little easy for things to just, well, slip completely out, so that in the evening you stay sitting and half-write and then you half-write a little like that throughout the day, so I think for my part I think it would have been a lot easier then with that structure, plus I do a lot of sports then. (11)

Several students mentioned problems with keeping their daily rhythm since they had to stay at their dorm or residential community most of the time:

Then it is that I was losing the routines quite quickly when the pandemic came – I thought this was rather hopeless, I would not be able to do this – but when I managed to maintain the routines, it was better to write the bachelor thesis. (7)

Since many of the students were stuck in small dormitories with only one room, some informants had to work on their bachelor’s thesis sitting on their bed, they had to eat, relax, and sleep in the same room, day and night. This situation disturbed their sleep patterns:

I have detected that, in a way, sitting in bed and working in it requires a lot of you, so to speak, both physically and psychologically – I sleep so badly when I sit in bed and work. (4)

Not only was sleep influenced by their situation, but it was also physically challenging when sitting in bed and writing on the thesis; the students may also experience pain in their back, as one of the students stated.

I also notice that my body hurts when sitting in an awkward position and writing. (1)

### Mental challenges

This category consists of statements that describe the mental challenges due to the COVID-19 pandemic, the overnight change in the students’ social environment, the feeling of isolation in small dorms or residential communities, and how the students described different solutions to cope with the isolation they experienced.

#### Social isolation and loneliness

Many of the students used to be very social before the pandemic, and they experienced the change to no social life as very difficult:

Yes, just finding motivation in the beginning, I think, was very difficult – there was a lot of talk about our situation, but it did not help at all suddenly from having a ‘super social’ environment to experiencing that everything is closed – you could not meet anyone. (10)

Some of the students also felt depressed during the first part of the isolation period; they experienced a lack of sleep and problems with falling asleep, as these students described. The students also described that the challenges brought about by the pandemic made them feel depressed and had trouble coping with the day and night:

At the beginning of the pandemic, I had turned around the clock – then I had to turn it back again – now I see the sun a bit and it helps the mood, and you do not get so depressed. (10)

Despite feeling isolated, the students were solution-oriented and described different approaches to cope with the study situation during the COVID-19 pandemic and the closing of society:

I want to learn, and also something that you can relate to practice, then it becomes a bit of extra motivation to get a little better or learn more, and then it’s not to stress too much, my other friends have been so incredibly stressed at times even though they’ve got almost 2 months left, then they’ve sat and cried in their room and then I’ve been a bit like that, you have plenty of time and you get good help and support and that it’s not necessary to maximise the crisis so early, it will probably be fine to discuss on the assignment online, so. (13)

Many students stated that they travelled home and lived with their parents for a period to avoid loneliness. Since the teaching was digital, it was unproblematic to carry on with the studies if they had access to the internet. On the other hand, it was also challenging since several had forgotten to bring enough literature:

I went home to my parents in the countryside, and then a lot of different things happened, which disrupted, you could say, the bachelor’s process, yes, so I was there for maybe 5–6 weeks before I went back again and I was able to concentrate and then I was more motivated by seeing others who had worked on the assignment, you had had greater access to books or the library and received help. (9)

Not all students had the opportunity to move with their parents, but they found other creative solutions for preventing loneliness while staying apart. Some established online chat groups acted as a substitute for not having the opportunity to socialise in person:

Yes, we have such a Skype group – not that we talk all the time, but we are on Skype together, and then you have something to wake up to – you must meet the rest of the group. We eat breakfast together, then we eat lunch together, and then we are in a way together with someone, even if we don’t have the microphone on when we work. It also becomes a little harder to take time off when you have an appointment. (7)

The students described different settings were working together with other students, some in pairs, and some in bigger groups. The digital groups became a substitute for ordinary social contact:

It would have been much easier to collaborate, to be able to read a little more of each other’s texts and in a way look at the structure a little more, it’s a little more difficult, now there will be messages back and forth with a few questions, but in a way it won’t be the same as sitting in a room together and being able to look at what we’ve written then. (2)

The students described that they used the informal social groups they already had established to ‘construct’ social meeting points on digital platforms. They were social together in another way, making it possible to discuss subjects related to their bachelor’s theses in these meetings.

#### Motivational and emotional challenges

The students described a lack of motivation due to the isolation and their situation as students:

I remember I had a day where I couldn’t get up, I just, I was kind of stressed, I didn’t feel like I was going to get anything done, and then I just lay in bed all day, and then I realised that I can’t do it, I have to, I have to finish, so I got up the next day and worked, but I want to say, yes, I think motivation has been particularly difficult during that period here. (10)

Being isolated while working on the bachelor’s thesis simultaneously made it difficult to stay focused and concentrated on their theses:

I have to say, the COVID-19 situation has had a powerful impact on me, I am dependent on working in the library, and being stuck in my room has made me unconcentrated and unfocused. It’s going pretty slow. (10)

On the other hand, their digital network groups acted as a motivator to keep working on their theses.

Yes, you are seen somehow and, not that anyone cares that you go, but in a way, you feel a small obligation then. (7)

The change in routines was a challenge to many of the students, and they also worried about finishing their bachelor’s theses in time. The students said it was important to find solutions to be able to continue writing the bachelor’s thesis and deliver it on time, even though it was demanding:

We have a group of girls where we discuss things and, just like that almost daily we meet and sort of bring up things we wonder about or just ask how they interpret it or think about things, so we work in a way with the same thing, even if we have different subjects, so yes I think that is a good help, and in a way, you get maybe a bit of motivation when you hear that others also have bad days, that it is not just yourself but also others and you may help and support each other. (8)

## Discussion

In this study, we wanted to explore how Norwegian undergraduate nursing students, writing their bachelor’s thesis, experienced coping with the impact of COVID-19 on their social and academic lives. The results showed that the lockdown was challenging for the students, and the pandemic affected their lives in both social and academic matters.

The close-down and isolation were very difficult to cope with, especially for those who went from a ‘super social’ environment to experiencing that everything was closed. Findings from other studies show that the COVID-19 outbreak had a great impact on people’s and students’ mental health and quality of life.^8,9,12,27^ The results from this study also confirm that the sudden lockdown disrupted the students’ usual lives profoundly.^3^ In this study, we also found that undergraduate nursing students experienced both practical and mental challenges like sleep deprivation, lack of motivation and loneliness during the pandemic, with a great impact on their social and academic lives. Some challenges may occur in the student population during pandemics that will happen in the future.

### Coping with a disrupted student life

The students in this study described different solutions to prevent isolation and loneliness. Since the social conditions were turned upside down in a short time, with almost no time to prepare, they were forced into a problem-solving state, but it did not change the situation. The students had no other alternative than to adapt to and cope with distance learning, as another study has also shown.^3^ Fear of infection transmission to the families or themselves may make coping challenging for the undergraduate nursing students.^4,8^ Nursing undergraduate students are expected to care of patients who are infected with the pandemic and can be at risk of being infected or bringing infection transmission to their families, both as students and after graduation.

The students in this study experienced it as hard to keep up with the changes the pandemic and close-down led to. Even if information were available on digital platforms, some informants described that they struggled with finding information and adjusting to the new situation. Another study also found that nursing students experienced doubt and helplessness as a reaction to a similar pandemic.^16^ Some students in this study experienced helplessness and had problems finding relevant support for writing their bachelor’s thesis. Some also described that they longed for the university to open and go back to normal. These findings are important for the universities to take into consideration to better facilitate students’ learning situations when deciding the balance between digital and physical courses, and during pandemic-like and lockdown situations.

The students in this study struggled with keeping routines and working on their bachelor’s theses. Keeping a daily rhythm is important for finishing the bachelor’s thesis, as other studies also show.^1,40^ Knowing that adolescents are a vulnerable group related to keeping up with routines, universities and societies need to facilitate structures for students to develop daily routines, and to enhance students’ learning, mental health and well-being.^41^ Even though some travelled home to their parents to avoid social isolation, several decided to return to the university city because it was difficult to concentrate and write the bachelor’s thesis at home. Experiencing that both efforts, traveling home and returning to the university, still made it difficult to concentrate and write the bachelor’s thesis, made some of the informants a bit frustrated. Another study found that being in a situation with lockdown and isolation may put one at risk of developing negative emotions.^3^ Universities must, in similar situations, prevent these negative effects by creating discussion groups, meeting points in digital platforms and such like.

### Mental challenges

The students in this study experienced pressure to finish their bachelor’s theses on time and to start working as nurses. However, the pandemic situation encouraged many nursing students to volunteer and help out in hospitals and nursing homes in addition to writing their bachelor’s theses.^42^ The need for volunteers and pressure to finish the bachelor’s theses was challenging, illustrating how a student’s life with double work might make a student more vulnerable to psychological stress.^13^ Some students handled the situation by talking to their fellow students, confirming the importance of active coping to handle mental health challenges during a crisis^43^ and being a nursing student in general.^41,44^

The students in this study described a lack of motivation to finish their bachelor’s thesis. When students are in danger of losing motivation, support from teachers is important.^45^ In the COVID-19 pandemic, the students were socially isolated from their teachers, making it harder to take an active role in the learning process, which is important for learning.^6,46^ Not all the students were aware of the opportunity to make digital contact with their teachers. Some students described that they formed groups of two or more students, that they ‘met’ every day on digital platforms, had meals together, and encouraged each other to work on their bachelor’s thesis. The establishment of digital groups illustrates that a problem-focused coping approach is helpful in situations where students are anxious or stressed.^13,43^ Research also shows that help-seeking is an important strategy, involving social interaction between students and teachers^47^ to achieve academic goals. Teachers must be aware of the possibility of a lack of motivation to finish their bachelor’s thesis among the undergraduate nursing students by making an educational programme with fixed and mandatory meeting points with all students, making information more easily accessible and emphasising motivation-enhancing factors, to name a few.

Our findings further indicate that the pandemic has increased differences among students regarding those who were social and those who were less social before the pandemic. Some of the informants described that they had no one to talk to, while others developed breakfast groups and discussed their progress with fellow students. The students with the least social network from before may have suffered the most during the social closure. Other studies have found that a lack of social interaction and contact with others may challenge a person’s ordinary coping strategies and how the person usually deals with problems.^15,16,43^

The students in this study described several challenges when writing their bachelor’s thesis during the COVID-19 pandemic, with closed universities. The pandemic hindered social contact and students’ abilities to physically meet and discuss the development of their bachelor’s thesis with other students. The lack of social interaction and contact may challenge one’s ordinary coping strategies. Other studies have pointed out that nursing students may undergo extreme psychological stress when facing a pandemic,^16^ they may develop negative coping strategies when faced with pressure^48^ and they might experience anxiety when writing their bachelor thesis.^49^ The students in this study described that after a while with the pandemic, they developed more positive problem-coping strategies. They managed to maintain their routines, for example, waking each other up and eating breakfast together online, and discussing their challenges in writing their bachelor’s theses.

The problem with sleeplessness, as described by the informants in this study, may become a serious problem for students. Sleep duration, problems with falling asleep, and sleeplessness are associated with several health-risk behaviours and poor academic achievement.^25^ Maintaining sleep patterns is therefore important for being able to work on the bachelor’s thesis.^26^ Students who experience sleeplessness, bad coping mechanisms, and violation of routines may be at risk of not fulfilling the academic achievement of delivering their theses and becoming a registered nurse.

Writing a bachelor’s thesis encourages the students to be aware of alternative ways of thinking,^50^ increase and deepen their knowledge regarding nursing care, and gain valuable knowledge that they can use in their professional practice as nurses.^51^ As such, the COVID-19 pandemic has brought many issues that are important to consider in the future of nursing education.^4,5^ Students who enter academic programmes in the future will begin their education aware of risks and challenges that no other cohort could have ever imagined, having witnessed the global recognition of the dedication, roles, and significant contributions that nurses play in the health care system.^51^

### Methodological considerations

One of the strengths of this study is the chosen methodology and the qualitative interviews aimed to recognise the sphere from the student’s viewpoints.^34^ The in-depth investigation generates an understanding of how Norwegian undergraduate nursing students, writing their bachelor’s thesis, experienced the impact of COVID-19 on their social and academic lives. Using a semi-structured method, the interview offered space for the informants to deepen their viewpoints and provide data on how and what they considered important in their situation. Dependability and confirmability are major elements in identifying the implications of this study, and much work was committed to exploring these topics.^35^ Interpretations of the data were frequently discussed among the authors to confirm the dependability of the result. A possible limitation of this study is that the findings may not be transferable to other settings and other undergraduate nursing students who started their education after the COVID-19 pandemic.

## Conclusion

This study showed that the COVID-19 pandemic influenced nursing students’ social and academic lives in several ways while the students worked on their bachelor’s theses. Experiencing social isolation, loneliness, and not being able to meet and discuss the challenges in writing the bachelor’s thesis with other students were stated as obstacles. Many students struggled with keeping their routines and being motivated to work on their bachelor’s theses. Some had problems with falling asleep and experienced sleeplessness, making it hard to keep up with their academic work. Therefore, it is important to develop strategies within the university that ensure that students have sufficient support to fulfil their academic achievement and deliver their bachelor’s theses on time. It is also important that universities develop strategies that support students in developing and maintaining social and educational relations. These findings may be used to shed light on other health students’ challenges during a pandemic, and the solutions can be easily transferred or developed further to other student groups.

## Supplemental Material

sj-docx-1-smo-10.1177_20503121251360296 – Supplemental material for Increased social differences, loneliness, and sleep deprivation among undergraduate nursing students in Norway, when writing their bachelor’s thesis during the COVID-19 pandemic. A qualitative study Supplemental material, sj-docx-1-smo-10.1177_20503121251360296 for Increased social differences, loneliness, and sleep deprivation among undergraduate nursing students in Norway, when writing their bachelor’s thesis during the COVID-19 pandemic. A qualitative study by Beate André, Guro Karlsholm and Kjersti Grønning in SAGE Open Medicine
