# Supplementary material for: Increased social differences, loneliness, and sleep deprivation among undergraduate nursing students in Norway, when writing their bachelor’s thesis during the COVID-19 pandemic. A qualitative study
Source: SAGE Open Med. 2025 Aug 23;13:20503121251360296. doi: 10.1177/20503121251360296 (PMC12375154; doi:10.1177/20503121251360296)
Supplement: sj-docx-1-smo-10.1177_20503121251360296 – Supplemental material for Increased social differences, loneliness, and sleep deprivation among undergraduate nursing students in Norway, when writing their bachelor’s thesis during the COVID-19 pandemic. A qualitative study [file sj-docx-1-smo-10.1177_20503121251360296.docx]

**Interview Guide**

Bakcground questions

- Type of thesis?
- Method used in thesis?
- Age?
- Previous Studies before entering nursing studies?
- Can you tell me about how you have worked on your bachelor's thesis so far?
- Can you describe a typical study session where you work on your bachelor's thesis?
- Is each session or day the same? If not, how do the days/sessions vary?
- How many hours do you work each day?
- Do you work only on weekdays or do all days (weekdays and weekends) blend together?
- Where is your workplace? (kitchen, bedroom, etc.)
- Do you work in the same place every day?
- How do you organize the subject matter or the important things you need to learn?
- How do you approach reading a scientific article?
  - Are you able to relate the content of the bachelor's thesis to previous learning from your studies?
- How do you keep your motivation during the work with the bachelor's thesis?
- How do you work when you lack motivation?
- What do you do when you get stuck?
- Who do you contact to ask something, or to discuss, when you need help?
- Do you have contact with fellow students to discuss questions related to the thesis?
- Is the work on the bachelor's thesis going as you expected, or have you discovered or established new work routines/ways of working?
- Do you have work methods you are satisfied with/think work well?
  - If yes, can you describe them?
- Will you have the same work strategy until submission or do you think you will change something?
- Do you enjoy writing your bachelor's thesis?
- What are your ambitions regarding grades?
- How has the collaboration with your supervisor been?
- Have you participated in seminars with other students?
  - Can you tell a little about your benefit from participating?
- Do you think it is best to write the thesis alone or do you wish to write in a pair or a small group?
  - Why?
- Is there anything you have found especially difficult in the process of writing the thesis?
- What would have been different if it hadn't been for Covid 19 and lock-down?
- What are your best tips for others who are writing a thesis and are in the same situation?
- Do you have any other thoughts or opinions about learning strategies when writing your bachelor's thesis that we haven't talked about?
